# Radiofrequency in Dermatosurgery

**DOI:** 10.4103/0974-2077.44168

**Published:** 2008

**Authors:** Sharad Mutalik

**Affiliations:** *Consultant Dermatologist, Skin and Cosmetology Clinic, Saaj Chambers, 562/5 Shivajinagar, Pune, India*

**Editorial Comments: Radiofrequency surgery**

The three articles on the use of radiofrequency pubished in this issue reflect the popularity of the technique of radiofrequency in India. The reasons are not difficult to seek – it is cheap, easy to handle, and needs little maintenance. With the introduction of several CO_2_ laser machines from China and other countries, the cost of CO_2_ laser machines has come down in recent times. Still, radiofrequency continues to be the preferred method for Indian dermatologists and has almost replaced electrosurgery.The main reason is that there is minimal collateral damage (up to 75 µm) caused by radiofrequency machines, making it superior to electrosurgery. Possible reasons for this minimal collateral damage are:

- There is minimal heating of tissue during the procedure.
- Only the tip of the electrode comes in contact with the tissue, that too for a very short time.
- The diameter of electrode is small and therefore, the electrode-tissue interface is minimized
- Radiofreqency is used at a high-frequency power, but at low intensity.

**It is important to remember some useful practical points while operating RF machines:**

1. The operator should remember to use the foot switch intermittently and not keep it pressed continuously; otherwise, the RF generator will get damaged.
2. To overcome the problem of controlling the pressure over the foot switch, now handpiece with fingertip switch is available, so that one has better control while operating.
3. Optimal power should be used. If the electrode sticks to the tissue while operating, it indicates that the power is insufficient. Gradually increase the power setting by 0.5 units until the electrode no longer sticks. On the other hand, if sparking or charring is seen, it indicates that the power is too high. Reduce the power setting appropriately till no charring is seen.
4. Pedunculated lesions may be clamped between two haemostats, cut with a wire loop, and base coagulated with a small ball tip electrode.
5. Small skin tags can be gripped with a toothed forceps and excised from the base with a wire loop electrode.
6. Moles and seborrheic keratoses can be shave-excised with a wire loop. A beginner should be careful while excising wide lesions.
7. A dermatologist should not attempt advanced procedures such as resurfacing and blepharoplasty unless adequately trained in this technique.
8. RF should not be done in the presence of oxygen as there is a risk of explosion.
9. The operator should consider wearing a surgical mask and eye protection when working on lesions containing HPV (human papilloma virus).
10. Prophylactic acyclovir or valacyclovir may be administered in cases with recent history of Herpes labialis (to be started a day prior to surgery and continued for one week), particularly if the procedure is to be performed on the face.
11. Informed consent should be taken in all cases.
12. It is contraindicated in patients with a cardiac pacemaker.
13. The handpiece and electrodes can be autoclaved or sterilised by using a formalin chamber or ethylene oxide. The electrodes may also be sterilised by keeping in glutaraldehyde solution (Cidex). Always refer to the manufacturer’s manual for details on sterilization.
14. Use a smoke evacuator while treating infective lesions.

